# The genome sequence of the caddisfly, *Mystacides azureus* (Linnaeus, 1761) (Trichoptera: Leptoceridae)

**DOI:** 10.12688/wellcomeopenres.27977.1

**Published:** 2026-09-28

**Authors:** David C. Lees, Witold Morek, Charlotte J. Wright, Dan Hall, Natural History Museum Genome Acquisition Lab, Darwin Tree of Life Barcoding Collective, Wellcome Sanger Institute Tree of Life Management, Samples and Laboratory team, Wellcome Sanger Institute Scientific Operations: Sequencing Operations, Wellcome Sanger Institute Tree of Life Core Informatics team, Tree of Life Core Informatics collective, Darwin Tree of Life Consortium

**Affiliations:** 1 Natural History Museum, London, England, UK; 2 Wellcome Sanger Institute, Hinxton, Cambridgeshire, England, UK

**Keywords:** Mystacides azureus, caddisfly, genome sequence, chromosomal, Trichoptera

## Abstract

We present a genome assembly from an individual female *Mystacides azureus* (caddisfly; Arthropoda; Insecta; Trichoptera; Leptoceridae). The genome sequence has a total length of 622.39 megabases. Most of the assembly (98.99%) is scaffolded into 20 chromosomal pseudomolecules, including the Z sex chromosome. The mitochondrial genome has also been assembled, with a length of 24.88 kilobases. This assembly was generated as part of the Darwin Tree of Life project, which produces genomes for eukaryotic species found in Britain and Ireland.

## Species taxonomy

Eukaryota; Opisthokonta; Metazoa; Eumetazoa; Bilateria; Protostomia; Ecdysozoa; Panarthropoda; Arthropoda; Mandibulata; Pancrustacea; Altocrustacea; Allotriocarida; Hexapoda; Insecta; Dicondylia; Pterygota; Neoptera; Eumetabola; Endopterygota; Aparaglossata; Panorpida; Amphiesmenoptera; Trichoptera; Integripalpia; Brevitentoria; Leptoceroidea; Leptoceridae; Leptocerinae; Mystacidini; *Mystacides*; *Mystacides azureus* (Linnaeus, 1761) (NCBI:txid446513)

## Background

The caddisfly *Mystacides azureus* (Linnaeus, 1761) is one of three species of *Mystacides* recorded in Britain and Ireland. Adults of all three species have conspicuous red eyes and strongly angled maxillary palps ( Barnard & Ross, 2012). *Mystacides azureus* and *M. nigra* are similar in appearance, but the wings of *M. azureus* have a bluish iridescence, whereas those of *M. nigra* are yellowish black; males can also be distinguished by their genitalia ( Wallace *et al.*, 2022). The third species, *M. longicornis*, is more readily distinguished by its banded brown wings ( Wallace *et al.*, 2022). *Mystacides azureus* and *M. nigra* are both known as black silverhorns, a name also used for *Athripsodes aterrimus* ( Mosely, 1939). The antennae of *M. azureus* are usually banded black and white, although a form with unbanded white antennae also occurs ( Wallace *et al.*, 2022).

*Mystacides azureus* has a Holarctic distribution ( Corallini & Cianficconi, 2007). Larvae of *Mystacides* occur in both running and still water and construct cases mainly from sand grains, often with added plant fragments ( Wallace *et al.*, 2003). Adults couple their wings during flight using minute hooks on the leading edge of the hindwing that engage with a groove in the forewing ( Mosely, 1921). Males have larger eyes than females and use their elongated maxillary palps to grasp the female’s abdomen during mating ( Corallini & Cianficconi, 2007; Petersson, 1987).

DNA barcode analysis contributed to the description of two new Japanese species in the *M. azureus* species group and indicated additional cryptic diversity within the group ( Kuhara *et al.*, 2023). The COI barcode recovered from the mitochondrial genome assembly presented in this genome note (accession OZ286740.1) matched BOLD:AAB1494. As accessed on 23 September 2026, BOLD reported a mean within-BIN uncorrected *p*-distance of 1.3% ( *n* = 72) and a maximum within-BIN *p*-distance of 3.37%. The minimum uncorrected *p*-distance to the nearest neighbouring BIN, BOLD:ACE0060, was also 3.37%. Both BINs contained records identified as *M. azureus*, with BOLD:ACE0060 represented by specimens from Iraq ( Ratnasingham *et al.*, 2024).

We present a chromosome-level genome sequence for *Mystacides azureus*, produced using the Tree of Life pipeline. The sequence is based on a specimen collected from Beinn Eighe National Nature Reserve, Scotland, UK (Figure 1). This assembly was generated as part of the Darwin Tree of Life Project, which aims to generate high-quality reference genomes for all named eukaryotic species in Britain and Ireland to support research, conservation, and the sustainable use of biodiversity ( Darwin Tree of Life Project Consortium, 2022).

As of September 2026, nine genome assemblies were available for the family Leptoceridae, and this assembly was one of two available for the genus *Mystacides*, according to NCBI Datasets ( O’Leary *et al.*, 2024). It is the RefSeq reference assembly for this species.

**Figure 1.**
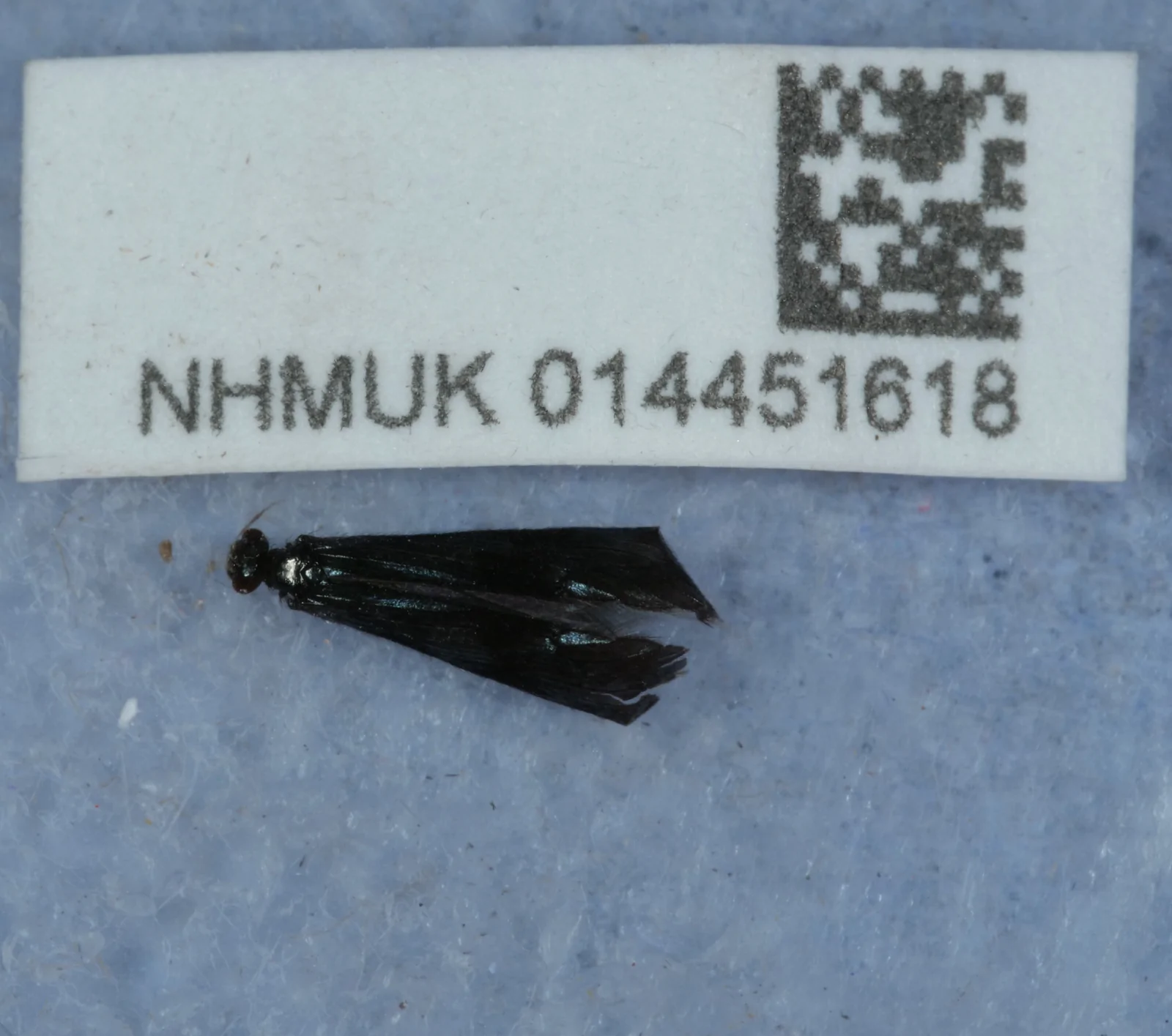
The *M. azureus* specimen (NHMUK014451618, iiMysAzur1) used for genome sequencing.

## Methods

### Sample acquisition and DNA barcoding

Two adult *Mystacides azureus* specimens were used: the female genome-sequencing specimen (specimen ID NHMUK014451618, ToLID iiMysAzur1; Figure 1) and the Hi-C and RNA specimen (specimen ID SAN00003319, ToLID iiMysAzur2). The genome-sequencing specimen was collected from Beinn Eighe National Nature Reserve, Scotland, UK (latitude 57.63, longitude −5.35) on 2021-09-09 by David Lees, who also identified it. The Hi-C and RNA specimen was collected from Milton Road, Cambridge, Cambridgeshire, England, UK (latitude 52.2259, longitude 0.1435) on 2023-06-29 by Witold Morek and identified by Charlotte Wright.

The initial identification was verified by an additional DNA barcoding process according to the framework developed by Twyford *et al.*. (2024). A small sample was dissected from the specimen and stored in ethanol, while the remaining parts were shipped on dry ice to the Wellcome Sanger Institute (WSI) (see the protocol). The tissue was lysed, the COI marker region was amplified by PCR, and amplicons were sequenced and compared to the BOLD database, confirming the species identification ( Crowley *et al.* , 2023). Following whole-genome sequencing, the relevant DNA barcode region was also used alongside the initial barcoding data for sample tracking at the WSI ( Twyford *et al.* , 2024). The standard operating procedures for Darwin Tree of Life barcoding are available on protocols.io.

### Nucleic acid extraction

High molecular weight (HMW) DNA extraction protocols developed by the Tree of Life Core Laboratory at the Wellcome Sanger Institute (WSI) are available on protocols.io ( Howard *et al.*, 2025). Tissue from the iiMysAzur1 sample was weighed and triaged to select the appropriate extraction workflow. For library DTOL13024303, tissue from the whole organism was homogenised using the PowerMash. HMW DNA was extracted in the WSI Scientific Operations core using the Automated MagAttract v2 protocol. DNA was sheared into an average fragment size of 12–20 kb following the Megaruptor®3 for LI PacBio protocol. Sheared DNA was purified by automated SPRI (solid-phase reversible immobilisation).

The concentration of the sheared and purified DNA was assessed with a NanoDrop spectrophotometer and a Qubit Fluorometer using the Qubit dsDNA High Sensitivity Assay kit. Fragment size distribution was evaluated by running the sample on the Femto Pulse system. For this sample, final post-shearing DNA measurements included a Qubit concentration of 9.6 ng/μL, a yield of 435.84 ng, and an average fragment size of 15.5 kb. The 260/280 spectrophotometric ratio was 2.61, and the 260/230 ratio was 2.32. The Genomic Quality Number (GQN) was 7.7.

RNA was extracted from abdomen tissue of the iiMysAzur2 specimen in the Tree of Life Laboratory at the WSI using the RNA Extraction: Automated MagMax™ *mir*Vana protocol. The RNA concentration was assessed with a NanoDrop spectrophotometer and a Qubit Fluorometer using the Qubit RNA Broad-Range Assay kit. RNA integrity was assessed using the Agilent RNA 6000 Pico Kit and Eukaryotic Total RNA assay.

### PacBio HiFi library preparation and sequencing

Library preparation and sequencing took place in the WSI Scientific Operations core. Library DTOL13024303 was prepared with the SMRTbell Prep Kit 3.0 (Pacific Biosciences) according to the manufacturer’s instructions. The kit includes reagents for end repair/A-tailing, adapter ligation, post-ligation SMRTbell bead clean-up, and nuclease treatment. Size selection and clean-up were performed using diluted AMPure PB beads (Pacific Biosciences). DNA concentration was quantified with a Qubit Fluorometer v4.0 (Thermo Fisher Scientific) and the Qubit 1X dsDNA HS assay kit. Final library fragment size was assessed with the Agilent Femto Pulse Automated Pulsed Field CE Instrument (Agilent Technologies) using the gDNA 55 kb BAC analysis kit.

The library was sequenced using the Sequel IIe system (Pacific Biosciences, California, USA). Library loading concentrations were in the range 40–135 pM. SMRT Link software (Pacific Biosciences) was used to configure and monitor the run and to perform primary and secondary data analysis.

### Hi-C

***Sample preparation and crosslinking***

Hi-C data were generated from 20–50 mg of the iiMysAzur2 head sample using the Arima-HiC v2 kit (Arima Genomics). Following the manufacturer’s instructions, tissue was fixed and DNA crosslinked using TC buffer to a final formaldehyde concentration of 2%. The tissue was homogenised using the Diagnocine Power Masher-II. Crosslinked DNA was digested with a restriction enzyme master mix, biotinylated, and ligated. Clean-up was performed with SPRISelect beads before library preparation. DNA concentration was measured with the Qubit Fluorometer (Thermo Fisher Scientific) and Qubit HS Assay Kit. The biotinylation percentage was estimated using the Arima-HiC v2 QC beads.

***Hi-C library preparation and sequencing***

Biotinylated DNA constructs were fragmented using a Covaris E220 sonicator and size-selected to 400–600 bp using SPRISelect beads. DNA was enriched with Arima-HiC v2 kit Enrichment beads. End repair, A-tailing, and adapter ligation were carried out with the NEBNext Ultra II DNA Library Prep Kit (New England Biolabs), following a modified protocol where library preparation occurs while DNA remains bound to the Enrichment beads. Libraries were amplified with KAPA HiFi HotStart mix and a custom Unique Dual Index (UDI) barcode set (Integrated DNA Technologies). Depending on sample concentration and biotinylation percentage determined at the crosslinking stage, libraries were amplified with 10–16 PCR cycles. Post-PCR clean-up was performed with SPRISelect beads. Libraries were quantified using the AccuClear Ultra High Sensitivity dsDNA Standards Assay Kit (Biotium) and a FLUOstar Omega plate reader (BMG Labtech).

Prior to sequencing, libraries were normalised to 10 ng/μL. Normalised libraries were quantified again to create equimolar and/or weighted 2.8 nM pools. Pool concentrations were checked using the Agilent 4200 TapeStation (Agilent) with High Sensitivity D500 reagents before sequencing. Libraries were sequenced on the Illumina NovaSeq X, generating paired-end 150-bp reads.

### RNA library preparation and sequencing

Libraries were prepared with the NEBNext® Ultra II Directional RNA Library Prep Kit for Illumina (New England Biolabs), following the manufacturer’s instructions. Poly(A) mRNA was isolated from 100 ng of total RNA using oligo(dT) beads, converted to cDNA, and uniquely indexed; 14 PCR cycles were performed. Libraries were prepared with an intended average fragment size of 100–300 bp. Libraries were quantified, normalised, and pooled equimolarly to a final concentration of 2.8 nM before dilution to 150 pM for loading. Sequencing was carried out on the Illumina NovaSeq X, generating paired-end reads.

### Genome assembly

Prior to assembly of the PacBio HiFi reads, a database of *k*-mer counts ( *k* = 31) was generated from the filtered reads using FastK. The *k*-mer frequency distribution was analysed with GenomeScope (version 2.1.0) ( Ranallo-Benavidez *et al.*, 2020), providing fitted point estimates of genome size, heterozygosity and repeat content.

The HiFi reads were assembled using Hifiasm ( Cheng *et al.*, 2021) with the --primary option. The Hi-C reads ( Rao *et al.* , 2014) were mapped to the primary contigs using bwa-mem2 ( Vasimuddin *et al.*, 2019), and the contigs were scaffolded in YaHS ( Zhou *et al.*, 2023) with the --break option for handling potential misassemblies. The scaffolded assemblies were evaluated using Gfastats ( Formenti *et al.* , 2022), BUSCO ( Manni *et al.*, 2021) and MerquryFK ( Rhie *et al.*, 2020).

The mitochondrial genome was assembled using MitoHiFi ( Uliano-Silva *et al.* , 2023). The MitoHiFi reference was the mitochondrial genome of *Triaenodes qinglingensis* (NC_069289.1).

### Assembly curation

The assembly was decontaminated using the Assembly Screen for Cobionts and Contaminants ( ASCC) pipeline. The flat files and maps for curation were generated with TreeVal. Manual curation was conducted primarily in PretextView and HiGlass ( Kerpedjiev *et al.* , 2018). Scaffolds were visually inspected and corrected as described by Howe *et al*. (2021). Manual corrections included 26 breaks, 63 joins, and removal of three haplotypic duplications. This reduced the scaffold count by 66.3%, increased the scaffold N50 by 10.4%, and reduced the total assembly length by 4.1%. The curation process is described at sanger-tol/curation-resources. A Hi-C contact map of the final assembly was generated with PretextSnapshot.

### Assembly quality assessment

The MerquryFK tool ( Rhie *et al.*, 2020) was run in a Singularity container ( Kurtzer *et al.*, 2017) to evaluate *k*-mer completeness and assembly quality for the primary and alternate haplotypes using the *k*-mer database ( *k* = 31) computed prior to genome assembly. The analysis outputs included assembly QV scores and completeness statistics.

The genome was analysed using the BlobToolKit pipeline, a Nextflow implementation of the earlier Snakemake version ( Challis *et al.*, 2020). PacBio reads were aligned to the assembly using minimap2 ( Li, 2018) and processed with SAMtools ( Danecek *et al.* , 2021) to generate coverage tracks. The pipeline runs BUSCO ( Manni *et al.*, 2021) using lineages identified from the NCBI Taxonomy ( Schoch *et al.*, 2020). For the three basal BUSCO lineages, eukaryota, bacteria and archaea, BUSCO genes are aligned to the UniProt Reference Proteomes database ( Bateman *et al.* , 2023) using DIAMOND BLASTp ( Buchfink *et al.*, 2021). The genome assembly is divided into chunks based on the density of BUSCO genes from the closest taxonomic lineage, and each chunk is aligned to the UniProt Reference Proteomes database using DIAMOND BLASTx. Sequences without DIAMOND BLASTx hits are extracted using seqtk and aligned to the NT database using BLASTn ( Altschul *et al.*, 1990). The outputs are consolidated into a BlobDir for visualisation in BlobToolKit. The BlobToolKit pipeline was developed using nf-core tooling ( Ewels *et al.* , 2020) and MultiQC ( Ewels *et al.*, 2016), with containerisation through Docker ( Merkel, 2014) and Singularity ( Kurtzer *et al.*, 2017).

## Genome sequence report

### Sequence data

PacBio sequencing of the *M. azureus* specimen generated 24.84 Gb (gigabases) from 2.04 million reads. We assembled the genome from these reads. GenomeScope analysis using a *p* = 2 model gave a *k*-mer-based haploid genome size estimate of 647.00 Mb, with heterozygosity of 0.94% and repeat content of 34.57%; the full-model fit was 98.68% (Figure 2). The GenomeScope estimates guided expectations for the assembly. The sequencing data provided approximately 37× coverage. Hi-C sequencing produced 115.72 Gb from 383.19 million reads, which were used to scaffold the assembly. RNA sequencing data were also generated and are available in public sequence repositories. Table 1 summarises the specimen and sequencing details.

**Figure 2.**
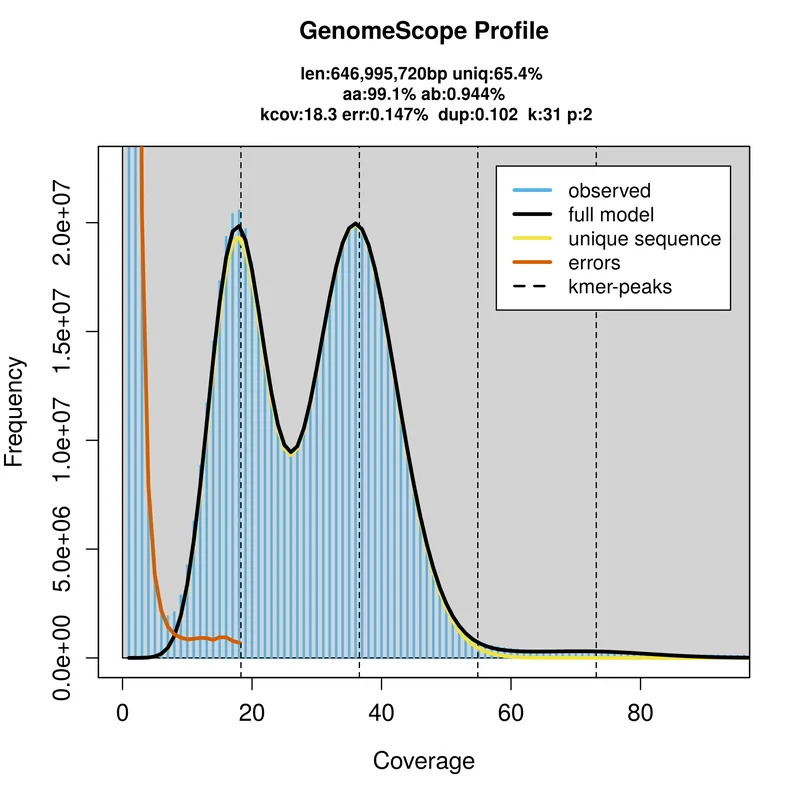
GenomeScope model fitted to the *k*-mer frequency distribution. The plot shows the observed and modelled *k*-mer spectra used to estimate genome size, heterozygosity and repeat content from unassembled sequencing reads.

**Table 1.** Specimen and sequencing data for *M. azureus* (BioProject PRJEB71288)

**Platform**	**PacBio HiFi**	**Hi-C**	**RNA-seq**
**ToLID**	iiMysAzur1	iiMysAzur2	iiMysAzur2
**Specimen ID**	NHMUK014451618	SAN00003319	SAN00003319
**BioSample (source individual)**	SAMEA14448314	SAMEA115163331	SAMEA115163331
**BioSample (tissue)**	SAMEA14448511	SAMEA115163477	SAMEA115163481
**Tissue**	whole organism	head	abdomen
**Instrument**	Sequel IIe	Illumina NovaSeq X	Illumina NovaSeq X
**Library ID & run accession(s)**	DTOL13024303: ERR12370421	ERR13925923	ERR13999090
**Read count total**	2.04 million reads	383.19 million read pairs	40.40 million read pairs
**Base count total**	24.84 Gb	115.72 Gb	12.20 Gb

### Assembly statistics

The primary haplotype was assembled, and contigs corresponding to an alternate haplotype were also deposited in INSDC databases. The final assembly has a total length of 622.39 Mb in 58 scaffolds, with 117 gaps, and a scaffold N50 of 31.37 Mb (Table 2).

**Table 2.** Genome assembly data for *M. azureus*

**Genome assembly**	**Primary assembly**
**Assembly name**	iiMysAzur1.1
**Assembly accession**	GCA_965644405.1
**Alternate haplotype accession**	GCA_965644555.1
**Assembly level**	chromosome
**Span (Mb)**	622.39
**Number of chromosomes**	20
**Number of contigs**	175
**Contig N50 (Mb)**	7.84
**Number of scaffolds**	58
**Scaffold N50 (Mb)**	31.37
**Sex chromosomes**	Z
**Organelles**	Mitochondrial genome: 24.88 kb

Most of the assembly sequence (98.99%) was assigned to 20 chromosome-level scaffolds, representing 19 autosomes and the Z sex chromosome. These chromosome-level scaffolds, confirmed by Hi-C data, are named according to size (Figure 3; Table 3).

The mitochondrial genome was also assembled (length 24.88 kb, OZ286740.1). This sequence is included as a contig in the multifasta file of the genome submission and as a standalone record.

**Figure 3.**
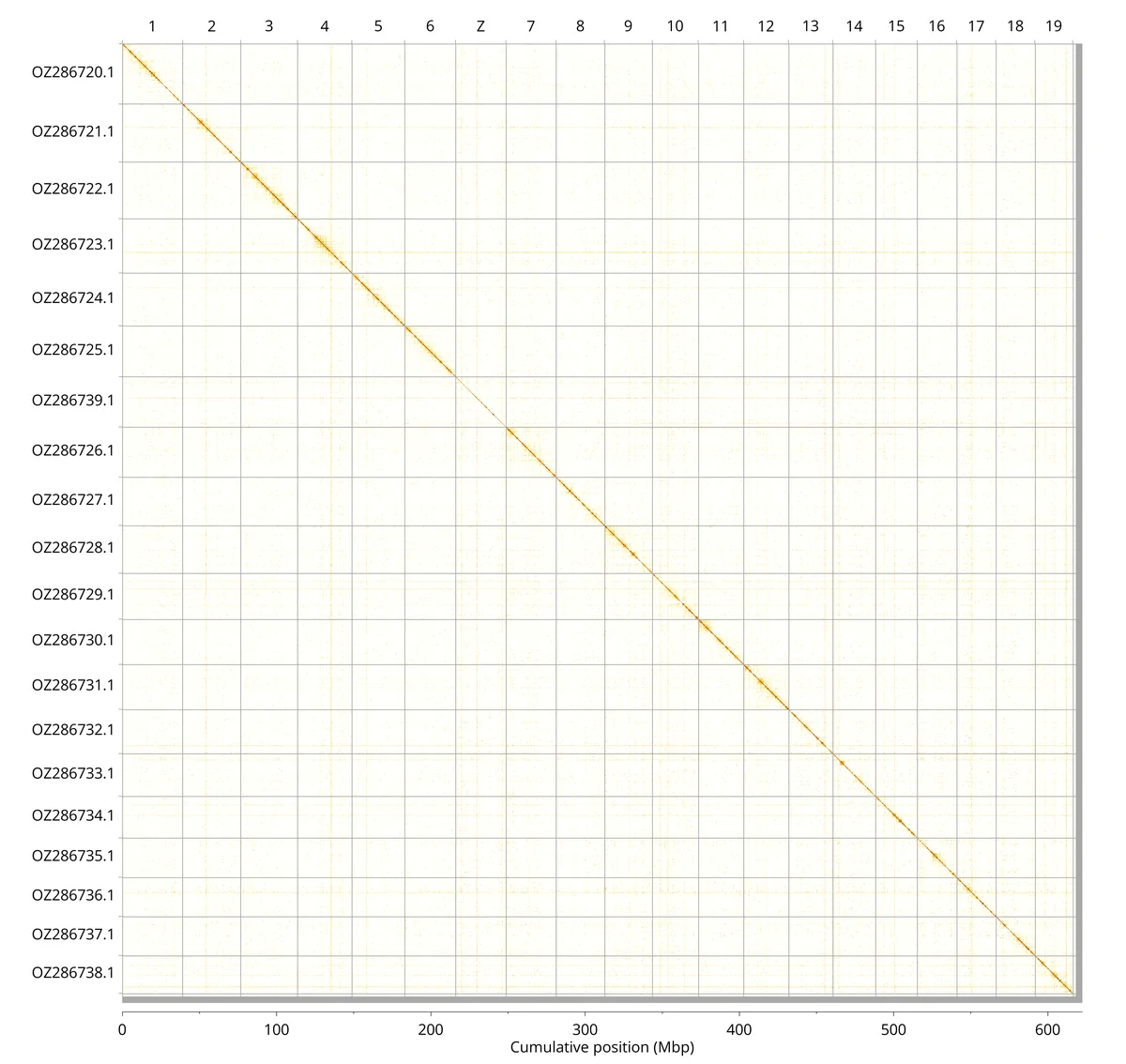
Hi-C contact map of the *M. azureus* genome assembly. Assembled chromosomes are shown in order of size and labelled along the axes, with a megabase scale shown below. The plot was generated using PretextSnapshot.

**Table 3.** Chromosomal pseudomolecules in the primary genome assembly of *M. azureus* iiMysAzur1

**INSDC accession**	**Molecule**	**Length (Mb)**	**GC%**
OZ286720.1	1	39.20	35.50
OZ286721.1	2	37.67	35
OZ286722.1	3	36.74	35
OZ286723.1	4	35.21	35
OZ286724.1	5	34.26	35
OZ286725.1	6	33	35
OZ286726.1	7	32.52	35
OZ286727.1	8	31.37	35
OZ286728.1	9	30.92	35.50
OZ286729.1	10	29.84	35.50
OZ286730.1	11	29.33	35
OZ286731.1	12	29.23	35
OZ286732.1	13	28.64	35.50
OZ286733.1	14	27.66	35.50
OZ286734.1	15	26.99	35.50
OZ286735.1	16	25.59	35.50
OZ286736.1	17	25.43	35.50
OZ286737.1	18	25.37	35.50
OZ286738.1	19	24.49	35.50
OZ286739.1	Z	32.66	35.50

### Assembly quality metrics

The combined primary and alternate assemblies achieve an estimated QV of 64.6. The *k*-mer completeness is 79.74% for the primary assembly, 76.73% for the alternate haplotype, and 98.85% for the combined assemblies (Figure 4).

**Figure 4.**
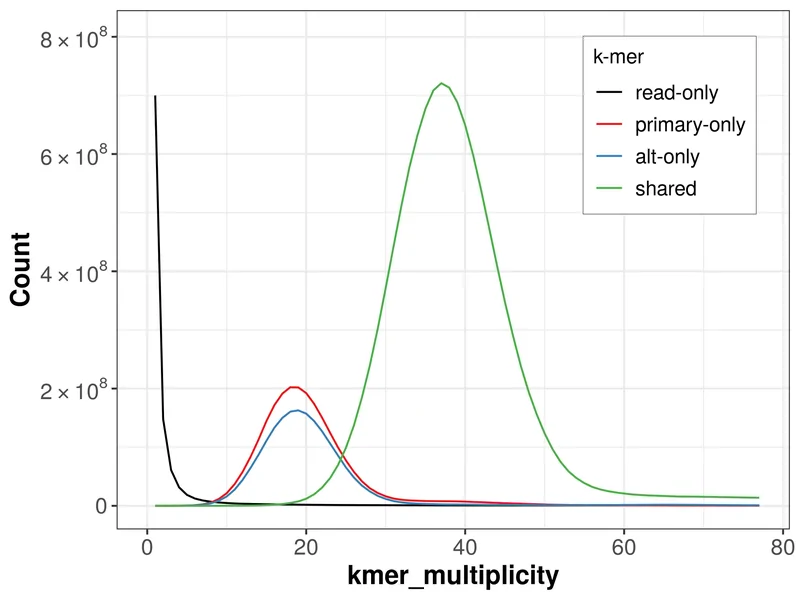
Evaluation of *k*-mer completeness using MerquryFK. This plot illustrates the recovery of *k*‐mers from the original read data in the final assemblies. The horizontal axis represents *k*‐mer multiplicity, and the vertical axis shows the number of *k*‐mers. The black curve represents *k*‐mers that appear in the reads but are not assembled. The green curve corresponds to *k*‐mers shared by both haplotypes, and the red and blue curves show *k*‐mers found only in one of the haplotypes.

BUSCO v.5.8.3 analysis using the endopterygota_odb10 reference set ( *n* = 2,124) identified 97.6% of the expected gene set (single = 96.4%, duplicated = 1.2%). The snail plot in Figure 5 summarises the scaffold length distribution and other assembly statistics for the primary assembly. The blob plot in Figure 6 shows the distribution of scaffolds by GC proportion and coverage.

**Figure 5.**
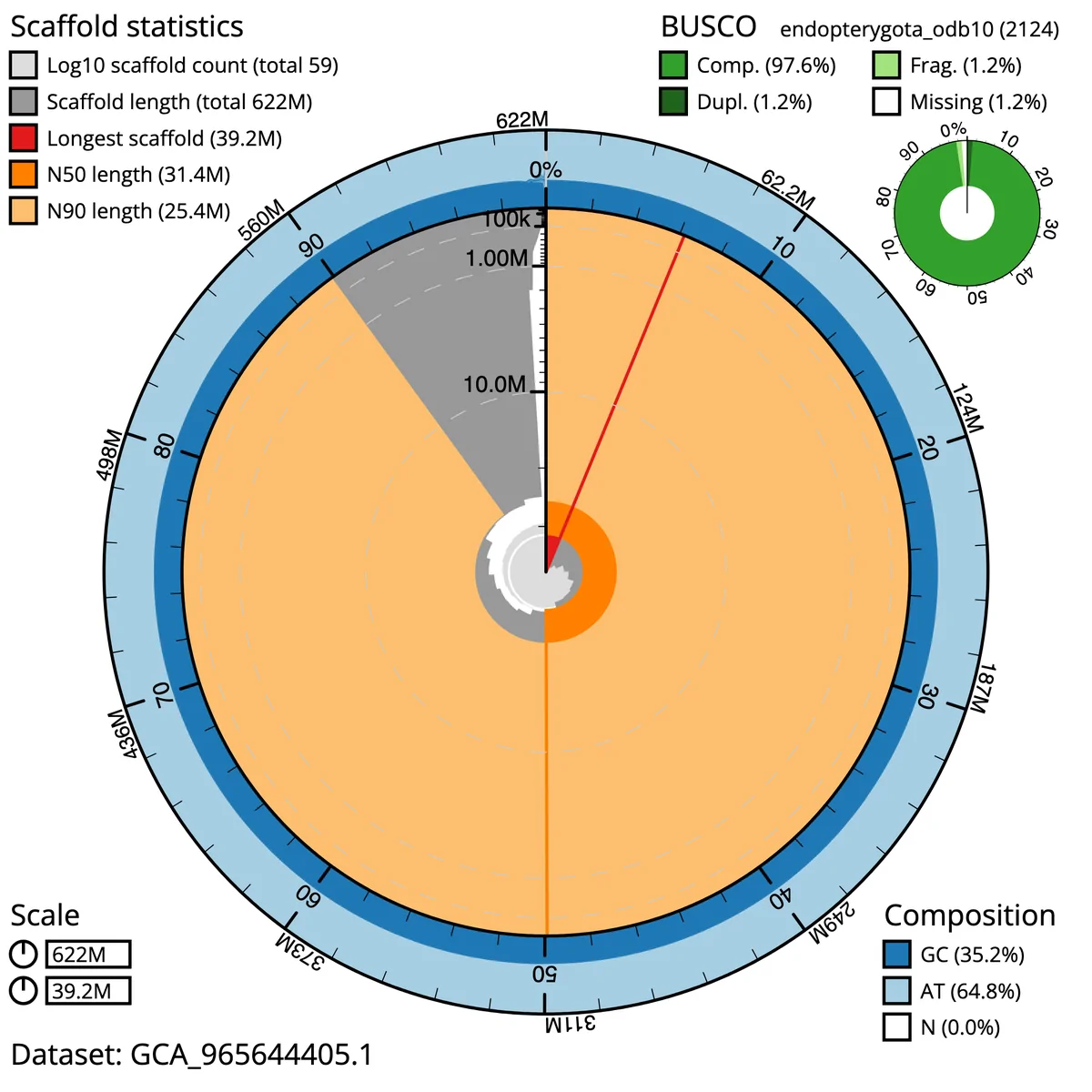
Assembly metrics for iiMysAzur1.1. The BlobToolKit snail plot provides an overview of assembly metrics and BUSCO gene completeness. The circumference represents the length of the whole genome sequence, and the main plot is divided into 1,000 bins around the circumference. The outermost blue tracks display the distribution of GC, AT, and N percentages across the bins. Scaffolds are arranged clockwise from longest to shortest and are depicted in dark grey. The longest scaffold is indicated by the red arc, and the deeper orange and pale orange arcs represent the N50 and N90 lengths. A light grey spiral at the centre shows the cumulative scaffold count on a logarithmic scale. A summary of complete, fragmented, duplicated, and missing BUSCO genes in the endopterygota_odb10 set is presented at the top right. An interactive version of this figure can be accessed on the BlobToolKit viewer.

**Figure 6.**
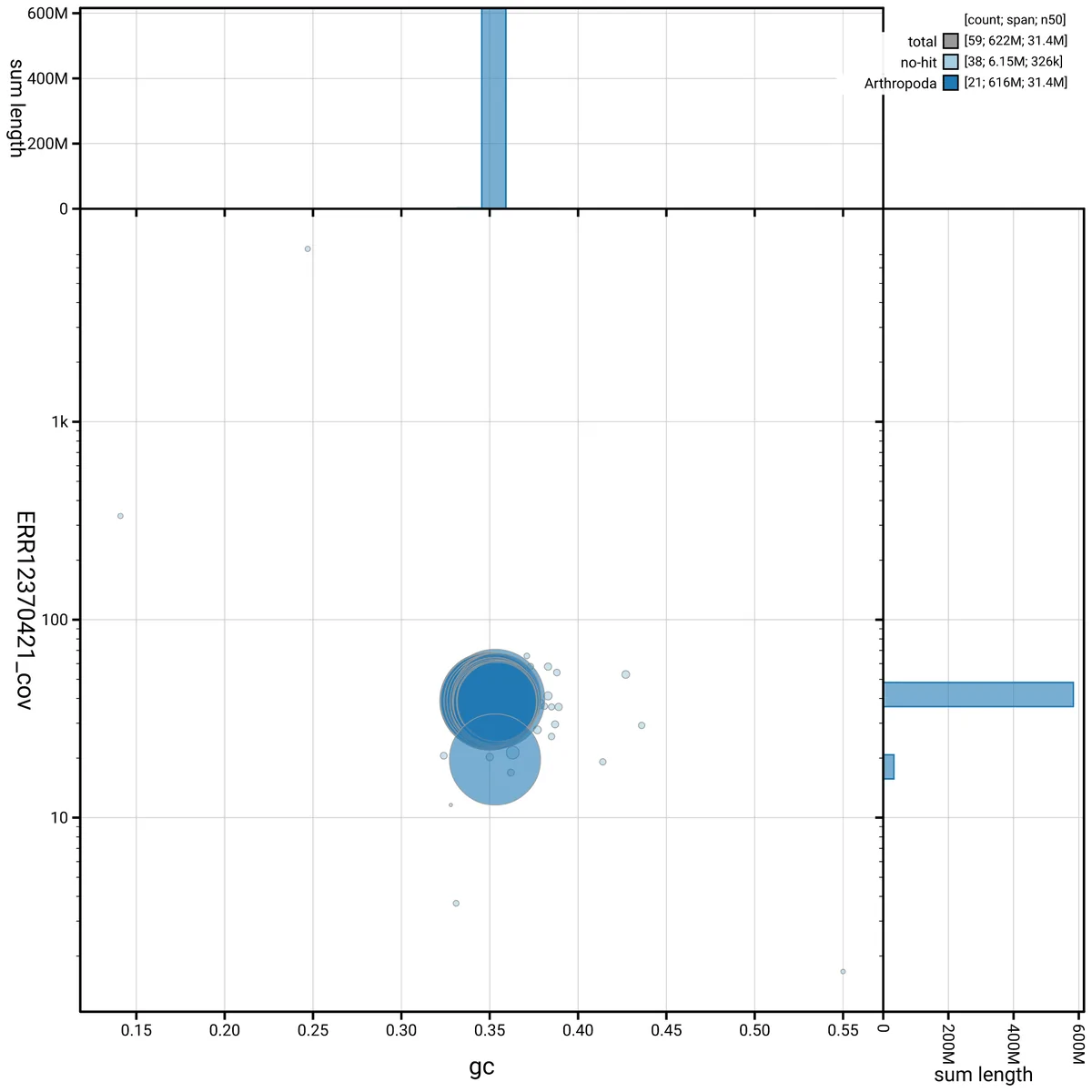
BlobToolKit blob plot for iiMysAzur1.1. The plot shows base coverage (vertical axis) and GC content (horizontal axis). The circles represent scaffolds, with the size proportional to scaffold length and the colour representing phylum membership. The histograms along the axes display the total length of sequences distributed across different levels of coverage and GC content. An interactive version of this figure is available on the BlobToolKit viewer.

Table 4 lists the assembly metric benchmarks adapted from Rhie *et al.*. (2021) and the Earth BioGenome Project (EBP) Report on Assembly Standards. The three-part EBP metric, calculated for the primary assembly, is **6.C.Q66**, meeting the 6.C.Q40 benchmark.

**Table 4.** Earth BioGenome Project summary metrics for the *M. azureus* assembly

**Measure**	**Value**	***Benchmark***
EBP summary (primary)	6.C.Q66	*6.C.Q40*
Contig N50 length	7.84 Mb	*> 1 Mb*
Scaffold N50 length	31.37 Mb	*= chromosome N50*
Consensus quality (QV)	Primary: 66.7; alternate: 63.9; combined: 64.6	*≥ 40*
*k*-mer completeness	Primary: 79.74%; alternate: 76.73%; combined: 98.85%	*> 90%*
BUSCO	C:97.6% [S:96.4%, D:1.2%], F:1.2%, M:1.2%, n:2,124	*S > 90%; D < 5%*
Percentage of assembly assigned to chromosomes	98.99%	*≥ 90%*

**Notes:** The EBP summary uses log10(Contig N50); chromosome-level (C) or log10(Scaffold N50); Q (Merqury QV). BUSCO v5.8.3 using the endopterygota_odb10 lineage dataset: C=complete; S=single-copy; D=duplicated; F=fragmented; M=missing; n=orthologues.

### Data availability

European Nucleotide Archive: Mystacides azureus, iiMysAzur. Accession number PRJEB71288; https://identifiers.org/ena.embl/PRJEB71288. The genome sequence is released openly for reuse. The *M. azureus* genome sequencing initiative is part of the Darwin Tree of Life Project ( PRJEB40665) and the Sanger Institute Tree of Life Programme ( PRJEB43745). All raw sequence data and the assembly have been deposited in INSDC databases. The genome will be annotated using available RNA-Seq data and presented through the Ensembl pipeline at the European Bioinformatics Institute. Raw data and assembly accession identifiers are reported in Table 1 and Table 2.

Production code used in genome assembly at the WSI Tree of Life is available at https://github.com/sanger-tol. Table 5 lists software versions used in this study.

**Table 5.** Software versions and sources used for *M. azureus*

**Software**	**Version**	**Source**
BLAST	2.14.0	https://ftp.ncbi.nlm.nih.gov/blast/executables/blast+/
BlobToolKit	4.4.6	https://github.com/genomehubs/blobtoolkit
blobtk	0.5.1	https://github.com/genomehubs/blobtk
BUSCO	5.8.3; 6.1.0	https://gitlab.com/ezlab/busco
bwa-mem2	2.2.1	https://github.com/bwa-mem2/bwa-mem2
DIAMOND	2.1.8	https://github.com/bbuchfink/diamond
fasta_windows	0.2.4	https://github.com/tolkit/fasta_windows
FastK	1.2	https://github.com/thegenemyers/FASTK
GenomeScope	2.1.0	https://github.com/tbenavi1/genomescope2.0
Gfastats	1.3.6	https://github.com/vgl-hub/gfastats
Hifiasm	0.19.8-r603	https://github.com/chhylp123/hifiasm
HiGlass	1.13.4	https://github.com/higlass/higlass
MerquryFK	1.1.0-c1	https://github.com/thegenemyers/MERQURY.FK
Minimap2	2.28-r1209	https://github.com/lh3/minimap2
MitoHiFi	3.2.2	https://github.com/marcelauliano/MitoHiFi
MultiQC	1.14; 1.17 and 1.18	https://github.com/MultiQC/MultiQC
Nextflow	24.10.4	https://github.com/nextflow-io/nextflow
PretextSnapshot	0.0.6	https://github.com/sanger-tol/PretextSnapshot
PretextView	1.0.3	https://github.com/sanger-tol/PretextView
samtools	1.21	https://github.com/samtools/samtools
sanger-tol/ascc	0.1.0	https://github.com/sanger-tol/ascc
sanger-tol/blobtoolkit	v0.8.0	https://github.com/sanger-tol/blobtoolkit
sanger-tol/curationpretext	1.4.2	https://github.com/sanger-tol/curationpretext
Seqtk	1.4-r122	https://github.com/lh3/seqtk
Singularity	3.9.0	https://github.com/sylabs/singularity
TreeVal	1.1.1	https://github.com/sanger-tol/treeval
YaHS	1.2a.2	https://github.com/c-zhou/yahs

### Author information

Contributors are listed at the following links:

- Members of the Natural History Museum Genome Acquisition Lab
- Members of the Darwin Tree of Life Barcoding collective
- Members of the Wellcome Sanger Institute Tree of Life Management, Samples and Laboratory team
- Members of Wellcome Sanger Institute Scientific Operations – Sequencing Operations
- Members of the Wellcome Sanger Institute Tree of Life Core Informatics team
- Members of the Tree of Life Core Informatics collective
- Members of the Darwin Tree of Life Consortium

### Wellcome Sanger Institute – Legal and Governance

The materials that have contributed to this genome note have been supplied by a Darwin Tree of Life Partner. The submission of materials by a Darwin Tree of Life Partner is subject to the **‘Darwin Tree of Life Project Sampling Code of Practice’**, which can be found in full on the Darwin Tree of Life website. By agreeing with and signing up to the Sampling Code of Practice, the Darwin Tree of Life Partner agrees they will meet the legal and ethical requirements and standards set out within this document in respect of all samples acquired for, and supplied to, the Darwin Tree of Life Project. Further, the Wellcome Sanger Institute employs a process whereby due diligence is carried out proportionate to the nature of the materials themselves, and the circumstances under which they have been/are to be collected and provided for use. The purpose of this is to address and mitigate any potential legal and/or ethical implications of receipt and use of the materials as part of the research project, and to ensure that in doing so we align with best practice wherever possible. The overarching areas of consideration are:

- Ethical review of provenance and sourcing of the material
- Legality of collection, transfer and use (national and international)

Each transfer of samples is further undertaken according to a Research Collaboration Agreement or Material Transfer Agreement entered into by the Darwin Tree of Life Partner, Genome Research Limited (operating as the Wellcome Sanger Institute), and in some circumstances, other Darwin Tree of Life collaborators.
