# Supplementary material for: The genome sequence of the caddisfly, Mystacides azureus (Linnaeus, 1761) (Trichoptera: Leptoceridae)
Source: Wellcome Open Res. 2026 Sep 28;11:780. [Version 1] doi: 10.12688/wellcomeopenres.27977.1 (PMC13687743; doi:10.12688/wellcomeopenres.27977.1)
Supplement: Peer review history

# Reviewer report: Reviewer response for version 1

**DOI:** 10.21956/wellcomeopenres.30779.r171338

**Published:** 2026-10-21

**Authors:** Natalia Florencio Martins

**Affiliations:** 1 Embrapa Agroindústria Tropical, Fortaleza, Brazil

This manuscript presents a high-quality, chromosome-level reference genome assembly for the caddisfly *Mystacides azureus* as part of the Darwin Tree of Life (DToL) Project. The authors generated long-read PacBio HiFi data, Omni-C/Hi-C data, and RNA-seq data using two separate individuals (iiMysAzur1 and iiMysAzur2). The primary assembly spans **622.39 Mb** across **20 chromosome-level scaffolds** (including the Z sex chromosome), with a scaffold N50 of **31.37 Mb** and a BUSCO completeness score of **97.6%**.

 In general, the manuscript is clear, the methodology is clear and the quality standards for indexing.

 Nevertheless, this reviewer propose few answers about the following topics:

 1. The GenomeScope k-mer profile estimated a haploid genome size of 647.00 Mb, whereas the final primary assembly spans 622.39 Mb. This ~24.6 Mb difference is modest (~3.8%) and likely attributable to collapsed repetitive elements.  Could the authors comment the stimative percentage of repetitive elements (LINES, SINES, etc)? 

 2. Did the cross-individual Hi-C mapping using iiMysAzur2 (collected in Cambridge) onto the iiMysAzur1 (collected in Scotland) long-read assembly encounter any notable structural polymorphism or inversion signals during manual curation in PretextView/HiGlass?

 3. About karyotypic comparison and sex determination, the assmbly resolved 20 chromosolam psudomolecules including Z chromosome. Since the sequence individue was declares as a female, was there any W chromosome saffold contig candidate? Is there a heterogametic sex in Trichoptera as WZ?

 4. About the mitochondrial genome, was the full mitochondrial gne order/syntheny verified against related Leptoceriadae species?

 In conclusion this reviewer recommends the approval of the manuscript and suggest   the authors to address  briefelly the questions above that  would enhance reader understanding regarding extraction parameters and sex chromosome resolution.

Are the datasets clearly presented in a usable and accessible format, and the assembly and annotation available in an appropriate subject-specific repository?

Yes

Are sufficient details of the sequencing and extraction, software used, and materials provided to allow replication by others?

Yes

Are the rationale for sequencing the genome and the species significance clearly described?

Yes

Are the protocols appropriate and is the work technically sound?

Yes

Reviewer Expertise:

Genomics, bioinformatics, protein structure.

I confirm that I have read this submission and believe that I have an appropriate level of expertise to confirm that it is of an acceptable scientific standard.

# Reviewer report: Reviewer response for version 1

**Published:** 2026-09-28

**Authors:** Automated Benchmarking

**Affiliations:** 1 Wellcome Sanger Institute, Hinxton, UK

As this article is a Genome Note, Automated Benchmarking has been used to determine whether the reported assembly passes four defined checks established by the Earth BioGenome Project: the raw and assembled data are publicly available, the Earth BioGenome Project three-part metric is met, the k-mer completeness exceeds 90%, and the BUSCO score passes the stated threshold. For Mystacides azureus (iiMysAzur1), all four checks passed. Data available: pass. ENA study PRJEB71288, raw-read accessions ERR12370421, ERR13925923, and ERR13999090, and assembly accessions GCA_965644405.1 and GCA_965644555.1 are public. Earth BioGenome Project three-part metric: pass. The primary assembly is reported as 6.C.Q66, meeting the 6.C.Q40 reference standard. k-mer completeness: pass. The combined haplotype assemblies have 98.85% k-mer completeness, exceeding the Earth BioGenome Project target of 90%. BUSCO: pass. The primary assembly, which is proposed as the reference assembly for this species, has 97.6% complete BUSCOs (96.4% single-copy and 1.2% duplicated). BUSCO software version: 5.8.3; lineage dataset: endopterygota_odb10. It meets the target of greater than 90% complete and single-copy BUSCOs. This decision uses values reported in the Genome Note and public accession records.

Reviewer Expertise:

NA

I confirm that I have read this submission and believe that I have an appropriate level of expertise to confirm that it is of an acceptable scientific standard.
